# Recombinant *Clostridium acetobutylicum* Endoxylanase for Xylooligosaccharide Production from Pretreated Lignocellulosic Biomass

**DOI:** 10.3390/biotech14040085

**Published:** 2025-10-30

**Authors:** Afifa Husna, Agustin Krisna Wardani, Chun-Yi Hu, Yo-Chia Chen

**Affiliations:** 1 Department of Biological Science and Technology, National Pingtung University of Science and Technology, Pingtung 912301, Taiwan; afifa.husna9@gmail.com; 2 Department of Agricultural Product Technology, Universitas Brawijaya, Malang 65145, Indonesia; agustinwardani@ub.ac.id; 3 Department of Food Science and Nutrition, Meiho University, Pingtung 912, Taiwan; x00011232@meiho.edu.tw

**Keywords:** *Clostridium acetobutylicum*, lignocellulosic biomass, xylooligosaccharides, xylan, xylanase

## Abstract

Xylooligosaccharides (XOS) are functional oligosaccharides with recognized prebiotic properties and growing industrial relevance, typically obtained through enzymatic depolymerization of xylan-rich lignocellulosic substrates. In this study, a recombinant endo-β-1,4-xylanase (XynA) from *Clostridium acetobutylicum* was employed for XOS production. The xynA gene was cloned into the expression vector pET-21a(+) and heterologously expressed in *Escherichia coli* BL21(DE3) under induction with isopropyl β-D-1-thiogalactopyranoside (IPTG). The recombinant protein, with an estimated molecular mass of 37.5 kDa, was verified by SDS-PAGE and Western blot analysis. Functional characterization via thin-layer chromatography revealed that XynA efficiently hydrolyzed beechwood xylan and rye arabinoxylan, predominantly yielding xylobiose. Additionally, the enzyme catalyzed the conversion of xylotriose into xylobiose and trace amounts of xylose. Notably, XynA demonstrated hydrolytic activity against autohydrolysed and alkali-pretreated coconut husk biomass, facilitating the release of XOS. These results underscore the potential of *C. acetobutylicum* XynA as a biocatalyst for the valorization of lignocellulosic residues into high-value oligosaccharides.

## 1. Introduction

Lignocellulosic agricultural residues such as oil palm shell (OPS), oil palm mesocarp (OPM), coconut husk (CH), and spent mushroom compost (SMC) represent abundant and underutilized biomass resources, particularly in tropical and subtropical regions where palm and coconut cultivation are economically dominant. These residues are often discarded or incinerated, contributing to environmental pollution and the release of greenhouse gases. However, their biochemical composition—primarily cellulose, hemicellulose, and lignin—offers considerable potential for valorization through biotechnological processes. The structural complexity and recalcitrance of lignocellulose pose challenges for direct utilization, necessitating targeted pretreatment strategies to unlock its biochemical potential. Globally, an estimated 140 billion metric tons of biomass are generated annually, much of which remains unexploited despite its renewability, biodegradability, and widespread availability [1,2]. Valorization of lignocellulosic biomass aligns with the principles of circular bio-economy and sustainable development, offering pathways to produce biofuels, platform chemicals, pharmaceuticals, and functional food ingredients. Among the major constituents of lignocellulose, hemicellulose is particularly rich in xylan—a structurally diverse heteropolysaccharide composed of β-1,4-linked xylose units with various side chains. Xylan can be enzymatically hydrolyzed to yield xylooligosaccharides (XOS), which have garnered attention for their prebiotic properties and health-promoting functions [3,4].

XOS are recognized for their ability to selectively stimulate the growth of beneficial gut microbiota such as Bifidobacterium and Lactobacillus, contributing to improved gastrointestinal health, enhanced calcium absorption, and reduced serum cholesterol levels [5,6,7,8]. These functional attributes have positioned XOS as a high-value ingredients in the food, nutraceutical, and pharmaceutical industries. However, commercial production of XOS remains limited by the cost and complexity of biomass processing, highlighting the need for efficient, scalable, and environmentally friendly conversion technologies.

Pretreatment is a critical step in the bioconversion of lignocellulosic biomass, aimed at disrupting the rigid matrix and enhancing the accessibility of hemicellulose. Among various strategies, autohydrolysis and alkaline extraction are widely employed due to their operational simplicity, low energy requirements, and minimal need for specialized equipment [9,10,11,12]. Autohydrolysis utilizes high-temperature water under pressure to solubilize hemicellulose, while alkaline pretreatment employs base solutions to cleave ester and ether linkages between lignin and carbohydrates. These methods are particularly suitable for decentralized biorefineries and agricultural processing facilities, where infrastructure and resources may be constrained. Following pretreatment, enzymatic hydrolysis of xylan into XOS is typically catalyzed by endo-β-1,4-xylanases (endoxylanases), which cleave internal glycosidic bonds within the xylan backbone. Microbial xylanases, especially those derived from bacteria, are preferred in industrial applications due to their thermostability, alkali tolerance, and robustness under harsh processing conditions. *Clostridium acetobutylicum* ATCC 824, a well-characterized anaerobic bacterium known for its solventogenic capabilities, has recently emerged as a promising xylan-degrading microorganism. Genomic analyses have revealed multiple xylanase-encoding genes within its genome, and the organism demonstrates the ability to grow on xylan substrates and produce short-chain oligosaccharides [13,14,15].

Despite its potential, the biochemical characteristics, substrate specificity, and catalytic efficiency of *C. acetobutylicum* xylanases remain insufficiently characterized. A deeper understanding of these enzymes is essential for optimizing their application in biomass conversion and tailoring their activity toward specific feedstocks. Moreover, the compatibility of these enzymes with pretreated tropical residues such as OPS, OPM, CH, and SMC has not been systematically evaluated, representing a critical gap in the development of regionally adapted bioprocesses.

In this study, OPS, OPM, CH, and SMC were subjected to autohydrolysis and alkaline pretreatment to extract crude hemicellulose enriched in xylan. The resulting extracts were used as substrates for XOS production via enzymatic hydrolysis with recombinant endoxylanase derived from *C. acetobutylicum* ATCC 824. This work aims to evaluate the efficiency of biomass pretreatment and enzymatic conversion, contributing to the valorization of agricultural residues through microbiologically informed biotechnological approaches. By integrating enzyme characterization with process optimization, this study supports the development of scalable platforms for XOS production from regionally abundant lignocellulosic feedstocks, advancing both microbial biotechnology and sustainable resource utilization.

## 2. Materials and Methods

### 2.1. Cloning and Expression of Endo-Xylanase from C. acetobutylicum

The DNA fragment encoding endo-1,4-β-xylanase (*xynA*) was amplified from the genomic DNA (gDNA) of *C. acetobutylicum* following the protocol described by Kramer and Coen [16]. Genomic DNA was extracted using the Tissue and Cell Genomic DNA Purification Kit (GeneMark, Taichung, Taiwan) according to the manufacturer’s instructions. PCR amplification was performed using the primers *xynA*-F (5′-CGCCGATCCGATAACCCAGTAGTGCAAACTCT-3′) and *xynA*-R (5′-CGGCTCGAGCTCTAGTTGATACAGTTTTTGTACCA-3′), incorporating *BamH*I and *Xho*I restriction sites, respectively. Polymerase chain reaction (PCR) amplification was performed under the following thermal cycling conditions: an initial denaturation step at 95 °C for 3 min; followed by 30 cycles comprising denaturation at 95 °C for 30 s, annealing at 56.7 °C for 30 s, and extension at 72 °C for 90 s; concluding with a final extension at 72 °C for 5 min. Amplified products were visualized by 0.8% (*w*/*v*) agarose gel electrophoresis and subsequently ligated into the pET21a(+) expression vector (Novagen) using standard cloning procedures. To confirm the successful ligation and correct orientation of the insert, the recombinant plasmid was subjected to DNA sequencing using T7 promoter and T7 terminator primers. Subsequently, the identity and domain architecture of the amplified *xynA* sequence were verified using the Basic Local Alignment Search Tool (BLAST+ 2.10.0; https://blast.ncbi.nlm.nih.gov/Blast.cgi (accessed on 1 April 2019)), allowing comparison with known xylanase sequences and confirmation of conserved catalytic motifs.

Protein expression was performed following the protocol described by Růčková et al. [17], with minor modifications. The recombinant plasmid pET-21a(+) carrying the *xynA* gene was introduced into *Escherichia coli* BL21(DE3) cells (Yeastern Biotech, New Taipei City, Taiwan) via heat-shock transformation. Transformed cells were cultured in LB medium at 37 °C until reaching an optical density at 600 nm (OD_600_) of approximately 0.6–0.8, at which point protein expression was induced by the addition of isopropyl-β-D-thiogalactopyranoside (IPTG) to a final concentration of 0.1 mM. Following induction, cells were harvested by centrifugation (6000× *g*, 10 min, 4 °C) and resuspended in lysis buffer (50 mM Tris-HCl, pH 8.0). Cell disruption was achieved by sonication on ice using a pulse cycle (10 s on, 20 s off) for a total of 5 min. The lysate was centrifuged (12,000× *g*, 15 min, 4 °C) to obtain the crude protein extract. The crude enzyme preparation was subsequently used to assess xylanase activity by incubating with various xylan-containing substrates to evaluate its capacity for XOS production.

### 2.2. SDS-PAGE and Western Blot Analysis

Recombinant protein expression following IPTG induction was examined by sodium dodecyl sulfate–polyacrylamide gel electrophoresis (SDS-PAGE) [18]. A total of 20 µg of crude protein extract was mixed with 2× sample buffer at a ratio of 2:1 (*v*/*v*) and loaded onto a polyacrylamide gel composed of a 10% resolving layer and a 5% stacking layer. Electrophoresis was performed in Tris-Glycine-SDS buffer at 90 V and 50 mA until adequate separation was achieved. Gels were subsequently stained overnight at 4 °C with gentle agitation using PageBlue™ Protein Staining Solution (Fermentas, Waltham, MA, USA), and destained the following day with multiple washes in deionized water to visualize protein bands.

For immunodetection [19], proteins were transferred from the gel onto an Immobilon-P polyvinylidene difluoride (PVDF) membrane (Millipore) using standard wet transfer procedures. Membranes were blocked for 15 min at room temperature in blocking buffer containing 1× phosphate-buffered saline with 0.05% Tween-20) and 3% (*w*/*v*) skim milk. After removal of the blocking solution, membranes were incubated overnight at 4 °C with mouse anti-His-probe monoclonal IgG1 antibody, followed by a 1-h incubation with goat anti-mouse secondary antibody at room temperature. Signal detection was performed using the BioSpectrum Imaging System (UVP, Upland, CA, USA).

### 2.3. Enzyme Activity and Substrate Specificity Assay

To evaluate the endo-xylanase activity of recombinant XynA, enzymatic assays were performed using a range of commercial polysaccharide substrates, including xylan from beechwood, oat spelt, and birchwood; xylooligosaccharides (XOS); wheat and rye arabinoxylan; β-glucan; avicel; lichenan; cellulose; and p-nitrophenyl-β-D-xylopyranoside (pNPX). Enzyme reactions were conducted by incubating XynA with each substrate at 60 °C for 10 min in a 1:1 ratio (*w*/*v*). The reactions were terminated by the addition of 3,5-dinitrosalicylic acid (DNS) reagent, followed by heating at 100 °C for 10 min. Reducing sugar release was quantified spectrophotometrically at 540 nm using a standard curve generated with xylose [20].

### 2.4. Hydrolysis Product Analysis

To confirm the generation of XOS with varying degrees of polymerization, thin-layer chromatography (TLC) was performed following the method of Cotta [21], with modifications. Enzyme–substrate mixtures were incubated at 60 °C for 24 h (1:1 ratio), and 1–2 µL of each hydrolysate was spotted onto TLC Silica Gel 60 F_254_ plates (Merck), alongside XOS and xylose standards (10 mg/mL). Plates were developed in a solvent system composed of ethyl acetate:acetic acid:formic acid:water (9:3:1:4, *v*/*v*) for 3 h. After development, plates were air-dried and sprayed with a visualization reagent consisting of methanol:sulfuric acid (6:1, *v*/*v*) containing 0.67 g methyl resorcinol monohydrate. Plates were then heated at 150 °C for 2 min to visualize oligosaccharide bands.

### 2.5. Biomass Selection and Pretreatment

Agricultural residues including oil palm mesocarp (OPM), oil palm shell (OPS), coconut husk (CH), and spent mushroom compost (SMC) were selected as substrates for evaluating the activity of recombinant endo-xylanase XynA. All biomass samples were air-dried, ground to a fine powder, and subjected to two distinct pretreatment protocols: autohydrolysis and alkaline extraction. Autohydrolysis was performed following the method described by Sabiha-Hanim et al. [22], with minor modifications. Briefly, 20 g of powdered biomass was suspended in distilled water at a ratio of 1:10 (*w*/*v*). The suspension was autoclaved at 121 °C under 1.2 kgf/cm^2^ pressure for 15 min. Post-treatment, the mixture was centrifuged at 2380× *g* for 15 min, and the supernatant was collected, filtered through Whatman No. 1 paper, and stored at −80 °C. Samples were subsequently freeze-dried for 72 h prior to enzymatic analysis. Alkaline pretreatment was conducted according to the protocol outlined by Hanim [23]. Biomass samples (20 g) were immersed in 3 M KOH solution at a ratio of 1:10 (*w*/*v*) and incubated at 40 °C for 4 h with gentle agitation. The resulting slurry was filtered, and the filtrate was acidified to pH 5.0–5.5 using 6 M HCl. The acidified solution was centrifuged at 3000 rpm for 15 min, and the pellet was dried at 60 °C for 24 h. Ethanol precipitation was performed by adding 95% ethanol to the supernatant in a 1:3 ratio (*v*/*v*), followed by centrifugation at 3000 rpm for 15 min. In this study, the soluble crude hemicellulose fraction (supernatant) was used for enzymatic hydrolysis with recombinant XynA to produce XOS, as it represents the fraction most accessible to enzymatic attack.

## 3. Results

### 3.1. Cloning and Expression of XynA from C. acetobutylicum

**Figure 1:**
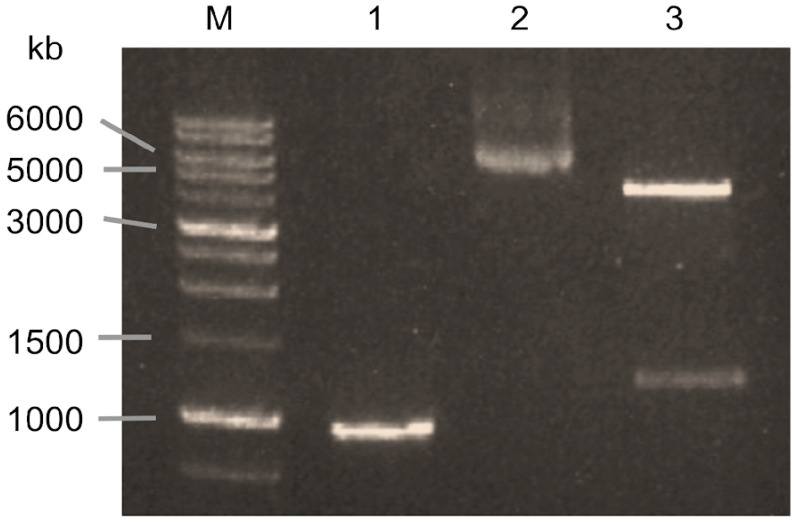
Agarose gel electrophoresis confirmation of pET21a-*xynA*. M = DNA marker, lane 1 = *xynA*, lane 2 = pET21a-*xynA*, lane 3 = digested pET21a-*xynA*.

**Figure 2:**
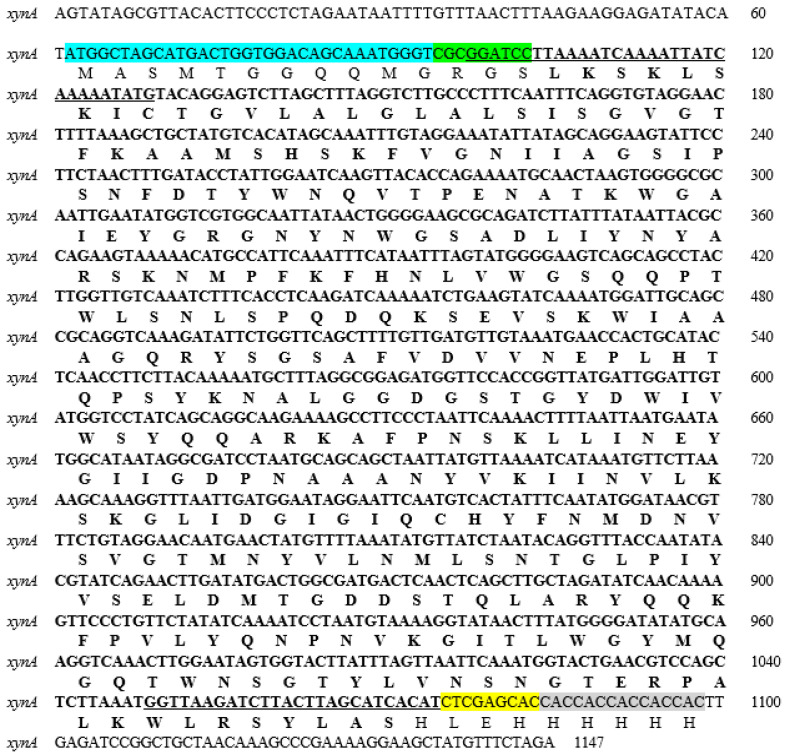
Nucleotide and deduced amino-acid sequence of *xynA* cloned from *C. acetobutylicum* using pET-21(a)+ vector. The forward and reverse primers for PCR amplification are underlined. The putative region is indicated in bold type. The fusion tags are highlighted in blue and grey for T7 and His Tag, respectively. The restriction sites are highlighted in green and yellow for the *BamH*I site and *Xho*I site, respectively.

The xynA gene was successfully cloned into the pET-21(a)+ expression vector (Figure 1) and subsequently verified through sequencing. Analysis of the nucleotide sequence revealed an open reading frame (ORF) of 957 bp, corresponding to a polypeptide of 318 amino acids. The deduced amino acid sequence was obtained using the BioEdit 7.2.5 software (Figure 2). BLAST analysis indicated that the encoded protein contains domains characteristic of the glycosyl hydrolase superfamily 1 and glycosyl hydrolase family 10. Furthermore, sequence alignment analysis revealed that the *xynA* gene product exhibits a high degree of sequence identity with characterized xylanases. Specifically, xynA shares 99.69% identity with endo-1,4-β-xylanase from *Clostridium acetobutylicum* ATCC 824, 89.31% identity with endo-xylanase from *Clostridium felsineum*, and 70.16% identity with β-xylanase from *Paenibacillus kobensis*. These results suggest a close evolutionary relationship between xynA and xylanases from *Clostridium* species, with comparatively lower similarity to the *Paenibacillus homolog*.

**Figure 3:**
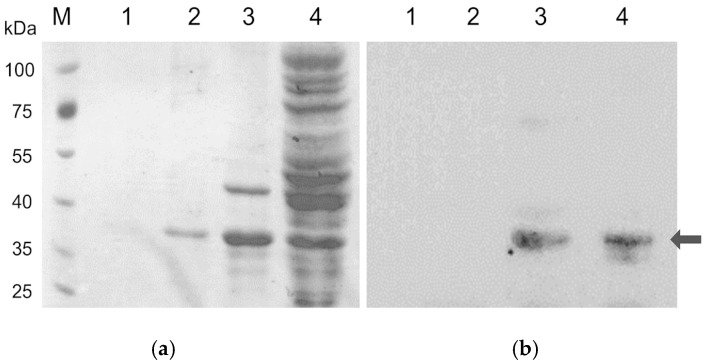
SDS-PAGE and Western blot analysis of XynA expression in *E. coli* BL21(DE3). XynA as protein target was indicated with arrow. (**a**) Crude protein extracts from *E. coli* BL21(DE3) transformed with the *xynA* expression construct were separated by SDS-PAGE. (**b**) Western blot analysis was performed using an anti-His antibody to detect the His-tagged XynA protein. Lane M: protein molecular weight marker; Lane 1: cell pellet prior to induction; Lane 2: cell lysate prior to induction; Lane 3: pellet after 16 h induction; Lane 4: cell lysate after 16 h induction.

The coding sequence of *xynA* was successfully amplified and inserted into the pET-21a(+) vector for heterologous expression, followed by transformation into *E. coli* BL21(DE3) cells. After induction of protein expression, the crude extract was analyzed by SDS-PAGE, revealing a prominent protein band of approximately 37.5 kDa (Figure 3). To confirm the identity of the expressed protein, a Western blot analysis was performed after 16 h of induction. The results (Figure 3) verified that the target protein, with a molecular weight of around 37.5 kDa, was present in both the soluble fraction and the cell pellet.

### 3.2. Endo-Xylanase Activity of XynA

The crude enzyme was evaluated for its substrate specificity using various polysaccharides, including oat spelt xylan, beechwood xylan, arabinoxylan from rye and wheat, β-glucan, avicel, and cellulose. As shown in Table 1, the enzyme exhibited the highest catalytic activity toward beechwood xylan, followed by notable activity against oat spelt xylan. It also demonstrated the ability to hydrolyze xylooligosaccharides (XOS). In contrast, no enzymatic activity was detected on non-xylan substrates such as β-glucan, avicel, and cellulose.

**Figure 4:**
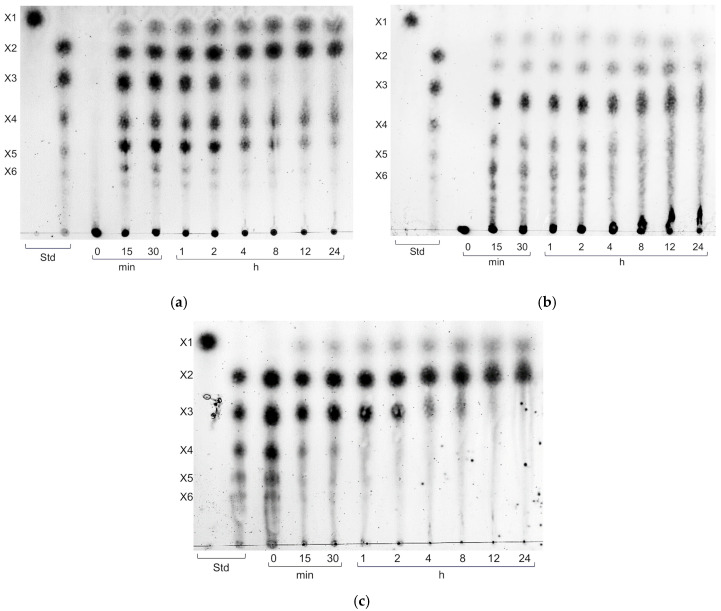
Thin-layer chromatography (TLC) analysis of XynA-mediated hydrolysis. TLC profiles of hydrolysates generated by XynA from beechwood xylan (**a**), rye arabinoxylan (**b**), and xylooligosaccharides (**c**) following incubation for 0 to 24 h.

TLC analysis (Figure 4) revealed that XynA efficiently hydrolyzed beechwood xylan into xylooligosaccharides (XOS; degree of polymerization [DP] 2–6) and monomeric xylose during the initial reaction phase (15 min to 2 h). Subsequently, xylotriose (X3) was further cleaved into xylobiose (X2) and xylose (X1), while xylohexaose (X6) was degraded into smaller oligosaccharides. Notably, xylotetraose (X4) and xylopentaose (X5) remained detectable throughout the 24-h incubation period, indicating partial resistance to complete hydrolysis. A similar degradation pattern was observed when XynA was incubated with purified XOS. Between 12 and 24 h, X3 was progressively hydrolyzed, yielding X2 and X1. In contrast, XynA exhibited limited activity toward rye arabinoxylan, failing to degrade X3 and higher DP XOS into smaller products even after 24 h of incubation.

These findings are consistent with the enzymatic profile of XynA as a typical endo-β-1,4-xylanase, exhibiting substrate specificity toward long-chain xylan polymers and preferentially cleaving internal β-1,4-glycosidic linkages.

### 3.3. Pretreatment of Agricultural Biomass and Enzymatic Hydrolysis by XynA

A range of agricultural residues, including oil palm mesocarp (OPM), oil palm shell (OPS), coconut husk (CH), and spent mushroom compost (SMC), were subjected to autohydrolysis and alkaline (KOH) pretreatment, followed by ethanol precipitation for crude hemicellulose extraction. As summarized in Table 2, autohydrolysis yielded lower amounts of crude hemicellulose compared to KOH treatment across all biomass types. Notably, for OPM and OPS, KOH pretreatment alone resulted in higher hemicellulose recovery than subsequent ethanol precipitation. In contrast, CH exhibited enhanced hemicellulose yield when KOH pretreatment was followed by ethanol precipitation.

**Figure 5:**
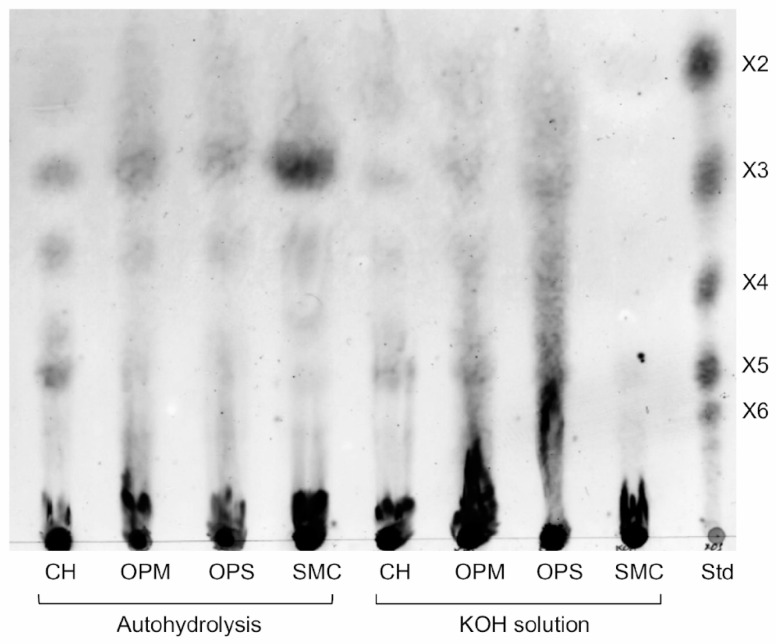
TLC analysis of XynA enzymatic products derived from various lignocellulosic substrates. TLC profiles of crude hemicellulose extracted from autohydrolyzed and KOH-pretreated substrates—coconut husk (CH), oil palm mesocarp (OPM), oil palm shell (OPS), and spent mushroom compost (SMC)—following hydrolysis by XynA.

The pretreated biomass samples were subsequently employed as substrates to evaluate the hydrolytic activity of XynA enzyme, specifically its ability to release xylooligosaccharides (XOS). As shown in Table 2, XynA exhibited substantial activity toward autohydrolyzed OPM and CH, as well as KOH-treated CH, OPM, and OPS. However, no detectable enzymatic activity was observed against SMC, regardless of the pretreatment method applied. These findings were corroborated by TLC analysis of XynA hydrolysis products derived from each biomass type (Figure 5), further confirming the substrate specificity and limited efficacy of XynA toward SMC-derived hemicellulose.

## 4. Discussion

Xylanases offer a sustainable alternative to chemical hydrolysis for the production of xylan-derived oligosaccharides, mitigating the environmental and safety concerns associated with harsh chemical treatments. These enzymes typically function as part of a synergistic consortium that includes endo-1,4-β-xylanase (E.C. 3.2.1.8), β-xylosidase (E.C. 3.2.1.37), α-glucuronidase (E.C. 3.2.1.139), α-L-arabinofuranosidase (E.C. 3.2.1.55), and acetylxylan esterase (E.C. 3.1.1.72), collectively facilitating the complete depolymerization of xylan into monomeric xylose [24]. Among these, endo-xylanase plays a pivotal role by cleaving internal β-1,4-glycosidic linkages within the xylan backbone, thereby generating xylooligosaccharides (XOS) as primary intermediates [25]. In this study, substrate specificity assays demonstrated that XynA exhibited hydrolytic activity exclusively toward substrates composed of β-1,4-linked xylose units, including beechwood xylan, oat spelt xylan, and XOS (Table 1). These results confirm that XynA functions as a typical endo-acting xylanase with strict specificity for linear xylan polymers. This preference for substrate is notably different from the wider range of specificity observed in a xylanase derived from *Clostridium acetobutylicum* ATCC 824, which has been demonstrated to act on a variety of polysaccharides and glycosidic substrates. These include carboxymethyl cellulose (CMC), avicel, lichenan, laminarin, polygalacturonic acid, β-glucan, and several p-nitrophenyl derivatives. The discrepancy in substrate range suggests potential evolutionary divergence in xylanase function among microbial taxa, possibly reflecting adaptation to distinct ecological niches or substrate availability. While further biochemical and structural analyses are warranted to elucidate the mechanistic basis of XynA’s specificity, the current findings reinforce its classification as a canonical endo-β-1,4-xylanase with limited promiscuity.

The hemicellulose yield obtained from KOH soaking followed by ethanol precipitation was lower than that from KOH soaking alone. This finding might be explained by the loss of low-molecular-weight hemicellulose fractions that remained soluble in ethanol during precipitation. Additionally, the filtration and washing steps performed after ethanol addition could have caused partial removal of soluble polysaccharides, particularly in oil palm mesocarp and shell, which contain heterogeneous and branched hemicellulose structures. Therefore, although ethanol precipitation is commonly applied to increase hemicellulose purity, it may also reduce total recovery depending on the molecular size distribution and solubility of the extracted polysaccharides.

Among the tested biomass sources, SMC yielded the highest amount of crude hemicellulose following pretreatment. This observation aligns with previous findings by Jurak et al. [26], who reported that xylan extracted via alkaline treatment from wheat-based compost used in *Agaricus bisporus* cultivation was heavily substituted with (4-O-methyl-)glucuronic acid and arabinosyl residues. Notably, both mono- and di-arabinosyl substitutions per xylosyl unit were retained. The persistence of these substituents is attributed to the limited enzymatic repertoire of *A. bisporus* during fruiting, particularly the absence of α-glucuronidase genes encoding GH115 enzymes capable of cleaving glucuronic acid side chains. Additionally, *A. bisporus* appears to lack the enzymatic machinery required for the removal of di-arabinosyl residues from xylosyl backbones [27].

Despite the high hemicellulose yield from SMC, XynA exhibited no detectable hydrolytic activity toward this substrate when pretreated with KOH, as evidenced by TLC analysis (Figure 5). In contrast, XynA effectively hydrolyzed other biomass types, releasing distinct xylooligosaccharides (XOS), including xylotriose (X3), xylopentaose (X5), and XOS resembling xylotetraose (X4). The lack of activity against SMC may be attributed to its heterogeneous composition, which includes not only lignocellulosic residues but also animal manure (e.g., horse or chicken), inorganic fertilizers, and calcium sulfate [28]. Given that crude hemicellulose was recovered via simple filtration, residual non-polysaccharide components may have co-extracted and acted as inhibitors of enzymatic activity. Further purification and compositional analysis of the hemicellulose fraction are warranted to confirm this hypothesis and assess its suitability for enzymatic hydrolysis.

TLC profiling of XynA hydrolysis products revealed that xylobiose (X2) and xylose (X1) were the predominant end products (Figure 4). Xylobiose, the shortest XOS comprising two β-1,4-linked xylose units, is particularly noteworthy due to its enhanced fermentability by gut microbiota and associated prebiotic benefits [29]. Moreover, short-chain XOS such as xylobiose possess favorable organoleptic properties, including a mild sweetness comparable to 30–40% of sucrose, as reported by Park et al. [30]. These attributes suggest that XynA-derived hydrolysates may hold promise for applications in functional food formulations and nutraceuticals.

## 5. Conclusions

The recombinant xylanase XynA (~37.5 kDa) exhibited robust hydrolytic activity toward xylan and arabinoxylan substrates, generating XOS as confirmed by TLC. In addition to its activity on purified polysaccharides, XynA effectively catalyzed the production of XOS from crude hemicellulose fractions derived from coconut husk, oil palm mesocarp, and oil palm shell following autohydrolysis and alkaline (KOH) pretreatment. These findings underscore the potential of XynA as a biocatalyst for valorizing agricultural residues into functional XOS.

## Figures and Tables

**Table 1:** Substrate specificity of XynA.

Substrate	Reducing Sugar Produced (mg/mL)
oat spelt xylan	1489.78 ± 3.33
beechwood xylan	1560.61 ± 3.21
rye arabinoxylan	1119.78 ± 26.13
wheat arabinoxylan	345.89 ± 12.88
XOS	291.72 ± 3.85
β-glucan	ND ^1^
CMC	ND
lichenan	ND
avicel	ND
cellulose	ND
p-Nitrophenyl Xylopiranoside	ND

^1^ ND means not detected.

**Table 2:** Hemicellulose yield and reducing sugar from pretreated biomass hydrolyzed by XynA.

Pretreatment Method	Biomass	Hemicellulose Yield (%)	Reducing Sugar Produced (mg/mL)
Autohydrolysis	OPM ^2^	4.37 ± 0.20	155.33 ± 10.09
	OPS ^3^	1.19 ± 0.18	93.67 ± 10.00
	CH ^4^	3.32 ± 0.25	179.78 ± 26.69
	SMC ^5^	8.90 ± 0.14	^1^ ND
KOH soaking	OPM	16.10 ± 0.58	77.00 ± 2.22
	OPS	3.32 ± 0.74	59.59 ± 4.49
	CH	5.30 ± 0.59	185.15 ± 9.45
	SMC	74.83 ± 0.71	ND
KOH soaking with ethanol precipitation	OPM	10.73 ± 0.28	299.22 ± 8.68
	OPS	1.84 ± 0.79	299.59 ± 10.26
	CH	16.10 ± 1.15	ND
	SMC	17.50 ± 0.51	ND

^1^ ND means not detected, ^2^ OPM means oil palm mesocarp, ^3^ OPS means oil palm shell, ^4^ CH means coconut husk, ^5^ SMC means spent mushroom compost.

## Data Availability

The original contributions presented in this study are included in the article. Further inquiries can be directed to the corresponding author.

## Abbreviations

The following abbreviations are used in this manuscript:

XOS	Xylooligosaccharides
TLC	Thin-Layer Chromatography
SDS-PAGE	Sodium Dodecyl Sulphate-Poly Acrylamide Gel Electrophoresis
IPTG	isopropyl β-D-1-thiogalactopyranoside
OPS	Oil Palm Shell
OPM	Oil Palm Mesocarp
CH	Coconut Husk
SMC	Spent Mushroom Compost
DP	Degree of Polymerization
CMC	Carboxyl Methyl Cellulose

## References

[1] Kumar S., Lohan S.K., Parihar D.S., Rakshit A., Biswas A., Sarkar D., Meena V.S., Datta R. (2023). Biomass energy from agriculture: Conversion techniques and use. Handbook of Energy Management in Agriculture.

[2] Kumari K., Singh A., Marathe D., Pariyar P., Khan A., Jawaid M., Pizzi A., Azum N., Asiri A., Isa I. (2021). Agricultural biomass as value chain developers in different sectors. Advanced Technology for the Conversion of Waste into Fuels and Chemicals.

[3] Kumari K., Nagar S., Goyal S., Maan S., Kumar V., Kharor N., Sindhu M., Kumar V. (2024). A fast, reliable, low-cost, and efficient xylan extraction for xylooligosaccharides production. Biofuels Bioprod. Bioref..

[4] Peralta A.G., Venkatachalam S., Stone S.C., Pattathil S. (2017). Xylan epitope profiling: An enhanced approach to study organ development-dependent changes in xylan structure, biosynthesis, and deposition in plant cell walls. Biotechnol. Biofuels.

[5] Kaprelyants L., Zhurlova O., Shpyrko T., Pozhitkova L. (2017). Xylooligosaccharides from agricultural by-products: Characterisation, production and physiological effects. Harčova Nauka Tehnol..

[6] Praveen K.G., Pushpa A., Prabha H. (2017). Value addition of orange fruit wastes in the enzymatic production of xylooligosaccharides. Afr. J. Biotechnol..

[7] Chaiyates R., Mahakhan P., Sawaengkaew J. (2024). Production of xylo-oligosaccharides from corncob using high efficiency xylanase from *Trichoderma harzianum* 4FR8. Biomass Conv. Bioref..

[8] Li T., Lei X., Wang L., Liu C., Qiu Q., Li Y., Song X., Xiong X., Zang Y., Qu M. (2023). Production of xylo-oligosaccharides with degree of polymerization 3–5 from wheat straw xylan by a xylanase derived from rumen metagenome and utilization by probiotics. Food Biosci..

[9] Carvalheiro F., Duarte L.C., Gírio F., Moniz P., Mussatto S.I. (2016). Hydrothermal/Liquid Hot Water Pretreatment (Autohydrolysis). Biomass Fractionation Technologies for a Lignocellulosic Feedstock Based Biorefinery.

[10] Hao N., Bezerra T.L., Wu Q., Ben H., Sun Q., Adhikari S., Ragauskas A.J. (2017). Effect of autohydrolysis pretreatment on biomass structure and the resulting bio-oil from a pyrolysis process. Fuel.

[11] Zhang W., You Y., Lei F., Li P., Jiang J. (2018). Acetyl-assisted autohydrolysis of sugarcane bagasse for the production of xylo-oligosaccharides without additional chemicals. Bioresour. Technol..

[12] Rigual V., Santos T.M., Domínguez J.C., Alonso M.V., Oliet M., Rodriguez F. (2018). Evaluation of hardwood and softwood fractionation using autohydrolysis and ionic liquid microwave pretreatment. Biomass Bioenerg..

[13] Ali M.K., Rudolph F.B., Bennett G.N. (2004). Thermostable xylanase10B from *Clostridium acetobutylicum* ATCC824. J. Ind. Microbiol. Biotechnol..

[14] Ali M.K., Rudolph F.B., Bennett G.N. (2005). Characterization of thermostable Xyn10A enzyme from mesophilic *Clostridium acetobutylicum* ATCC 824. J. Ind. Microbiol. Biotechnol..

[15] Lee S.F., Forsberg C.W., Gibbins L.N. (1985). Xylanolytic activity of *Clostridium acetobutylicum*. Appl. Environ. Microbiol..

[16] Kramer M.F., Coen D.M. (2001). Enzymatic amplification of DNA by PCR: Standard procedures and optimization. Curr. Protoc. Cell Biol..

[17] Růčková E., Müller P., Vojtěšek B. (2014). Protein expression and purification. Klin. Onkol..

[18] Laemmli U.K. (1970). Cleavage of structural proteins during the assembly of the head of bacteriophage T4. Nature.

[19] Mahmood T., Yang P.C. (2012). Western blot: Technique, theory, and trouble shooting. N. Am. J. Med. Sci..

[20] Miller G.L. (1959). Use of dinitrosalicylic acid reagent for determination of reducing sugar. Anal. Chem..

[21] Cotta M.A. (1993). Utilization of xylooligosaccharides by selected ruminal bacteria. Appl. Environ. Microbiol..

[22] Sabiha-Hanim S., Noor M.A.M., Rosma A. (2011). Effect of autohydrolysis and enzymatic treatment on oil palm (*Elaeis guineensis* Jacq.) frond fibres for xylose and xylooligosaccharides production. Bioresour. Technol..

[23] Nasir M.A.M., Saleh S.H. (2016). Characterization of hemicelluloses from oil palm empty fruit bunches obtained by alkaline extraction and ethanol precipitation. Malays. J. Anal. Sci..

[24] Chakdar H., Kumar M., Pandiyan K., Singh A., Nanjappan K., Kashyap P.L., Srivastava A.K. (2016). Bacterial xylanases: Biology to biotechnology. 3 Biotech.

[25] Santos C.R., Hoffmam Z.B., de Matos Martins V.P., Zanphorlin L.M., De Paula Assis L.H., Honorato R.V. (2014). Molecular mechanisms associated with xylan degradation by Xanthomonas plant pathogens. J. Biol. Chem..

[26] Jurak E., Patyshakuliyeva A., Kapsokalyvas D., Xing L., Van Zandvoort M.A.M.J., De Vries R.P., Gruppen H., Kabel M.A. (2015). Accumulation of recalcitrant xylan in mushroom-compost is due to a lack of xylan substituent removing enzyme activities of *Agaricus bisporus*. Carbohydr. Polym..

[27] Jurak E., Patyshakuliyeva A., De Vries R.P., Gruppen H., Kabel M.A. (2015). Compost grown *Agaricus bisporus* lacks the ability to degrade and consume highly substituted xylan fragments. PLoS ONE.

[28] Kapu N.U.S., Manning M., Hurley T.B., Voigt J., Cosgrove D.J., Romaine C.P. (2012). Surfactant-assisted pretreatment and enzymatic hydrolysis of spent mushroom compost for the production of sugars. Bioresour. Technol..

[29] Zhao S., Lau R., Chen M.H. (2024). Influence of chain length on the colonic fermentation of xylooligosaccharides. Carbohydr. Polym..

[30] Park H.W., Kim M.J., Seo S., Yoo S., Hong J.H. (2017). Relative sweetness and sweetness quality of xylobiose. Food Sci. Biotechnol..

